# The Use of Screening Algorithm to Defer Blood Donors with Subclinical Malaria

**DOI:** 10.1155/2021/9942721

**Published:** 2021-08-13

**Authors:** Desmond Omane Acheampong, Enoch Aninagyei

**Affiliations:** ^1^Department of Biomedical Sciences, School of Allied Health Sciences, College of Health and Allied Science, University of Cape Coast, Cape Coast, Ghana; ^2^Department of Biomedical Sciences, School of Basic and Biomedical Sciences, University of Health and Allied Sciences, PMB 31, Ho, Volta Region, Ghana

## Abstract

*Plasmodium falciparum* infection in blood donors is common in malaria endemic countries, including Ghana. To date, there are no established exclusion criteria to defer a donor carrying malaria parasites. Therefore, based on significant independent variables identified in this study, donor malaria screening algorithm was developed to be used by blood banks to screen blood donors for subclinical malaria. Each significant variable was weighted one (1) point and its alternative response was weighted negative one (−1) point. Accumulation of the points determines the risk level of the donor. These weighted points were used to categorize infected donors as having negligible (<2 points), tolerable (3-4 points), undesirable (5–8 points), or intolerable (>9 points) risk. Based on accumulated weight of ≥5 points, the algorithm was 94.7% (54/57) sensitive but 82% (298/364) specific. With this level of specificity, 18% of the blood donors without malaria would have been deferred. Therefore, it is imperative that all donors with accumulated risk ≥5 be screened for malaria using either malaria rapid test kit or microscopy.

## 1. Introduction

The common mode of transmission of *Plasmodium* spp. is through the injection of sporozoites by female *Anopheles* mosquitoes [1]. However, other modes of transmission, such as through blood transfusion [2], organ transplantation, and needle stick injury [3], have also been established. Transfusion-transmitted malaria (TTM) is an incidental transmission of *Plasmodium* parasite from asymptomatic donors to blood recipients. TTM is more serious in blood recipients from malaria nonendemic countries and in blood recipients in endemic countries with no or partial antimalaria immunity [4]. *P. falciparum* is commonly implicated in TTM followed by *P. malariae*, *P. vivax*, and *P. ovale* [2].

In sub-Saharan Africa, the prevalence of *Plasmodium* spp. parasitemia in blood donors ranges from 0.6% to 50.0% [5]. In addition, in Thailand, microscopic prevalence of *Plasmodium* spp. in blood donors was 0.27%, while submicroscopic prevalence was 2.33% [6]. In Ghana, varying prevalence of *P. falciparum* in blood donors has been reported from some of the regions. In the Volta region, a prevalence of 10.0% was reported [7], while in the Ashanti region, two studies reported a prevalence of 8.0% [8] and 13.0% [9]. In 2019, the prevalence of *P. falciparum* in blood donors screened in the Greater Accra Region was reported as 3.2% [10]. Finally, in the central region, a prevalence of 6.2% has also been reported [11]. Despite these reports, blood donors in most malaria endemic countries including Ghana are not routinely screened for malaria [12], in spite of WHO recommendations that all blood donors should be screened as such [13].

Lack of screening protocols for malaria in blood donors has resulted in the banking of a sizeable amount of infected donor blood. Despite the unavailability of sensitive malaria diagnostic techniques to detect low levels of the parasites in blood donors, microscopy and malaria rapid diagnostic techniques have been able to detect donors with asymptomatic malaria. Hence, deferral of infected donors has been recommended [14]. In spite of numerous reports on the occurrence of *P. falciparum* in blood donors, no study has reported on the demographic and transmission factors associated with *P. falciparum* infections in blood donors and exclusion criteria for deferment in Ghana and elsewhere. Therefore, this study was designed to identify exclusion criteria to be used to defer blood donations from donors with subclinical malaria.

## 2. Materials and Methods

### 2.1. Study Design, Study Sites, and Study Duration

**Figure 1:**
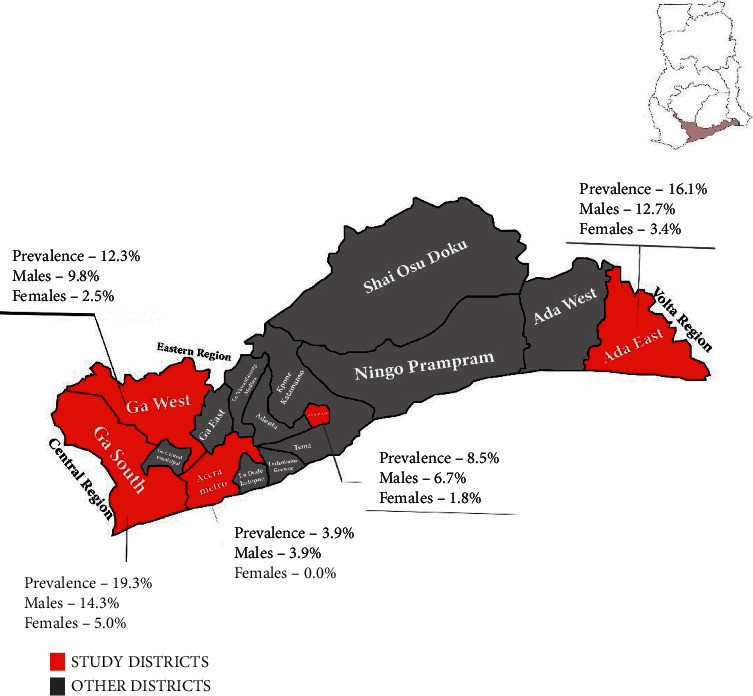
Distribution of *P. falciparum* infections in blood donors in Greater Accra Region, Ghana (the map is the authors' own production).

This study was a case-control study that sought to determine factors associated with subclinical malaria in voluntary nonremunerated blood donors. Blood donors were recruited from five randomly selected districts in Greater Accra Region of Ghana. The districts were Ada East (5°47′0″N, 0°38′0″E), Ashaiman (5°42′0″N, 0°2′0″W), Accra Metro (5°33′0″N, 0°12′0″W), Ga West (5°42′9″N, 0°18′0″W), and Ga South (5°34′0″N, 0°20′0″W) (Figure 1). Donor samples were collected from March to November 2018.

### 2.2. Inclusion and Exclusion Criteria

Prospective blood donors included in this study were individuals that satisfied the criteria for blood donation in Ghana and had stayed continuously in the respective district for over a year. In Ghana, eligible donors satisfy the following criteria: age 19–60 years, hemoglobin >12.5 g/dL, body weight >50 kg, systolic pressure 120–130 mmHg, and diastolic 70–80 mmHg. Consent to participate in this study was crucial. Excluded donors were those with successful phlebotomy but were unable to provide responses to the study questions and vice versa. Additionally, donors found to be infected with either HIV, *T. pallidum,* or hepatitis B or C virus were excluded from the study.

### 2.3. Number of Donor Blood Samples Collected

The overall prevalence of *P. falciparum* infection in Ghanaian blood donors studied in multicenters in Greater Accra Region of Ghana is not available so the prevalence was estimated as 50%. The sample size was calculated to be 384 using Cochrane's formula, *n* = *z*^2^*p*(1 − *p*)/*d*^2^, where *n* is the sample size, *z* is confidence level at 95% (standard value of 1.96), and *d* is margin of error at 5% (standard value of 0.05). Based on this sample size, the minimum number of blood donors sampled in each of the five districts was 76. The minimum number of voluntary donors to be recruited from each study site was found to be attainable due to the knowledge of the number of voluntary blood donors that was bled in 2016. In 2016, the numbers of voluntary blood donors that were bled in the five districts were as follows: Ada East *n* *=* 336, Ashaiman *n* *=* 307, Accra Metro *n* *=* 411, Ga West *n* *=* 241, and Ga South *n* *=* 216 (source: *District Donor Registration Book*). In the current study, blood donors that consented to partake in this study were randomly recruited, using stratified random sampling. Five districts were randomly selected and within each district, donors were randomly selected by systematic sampling where every 2nd donor with consent was recruited.

### 2.4. Donor Phlebotomy and Study Specimen Collection

Donor phlebotomy was done according to guidelines on drawing blood published by World Health Organization [15]. Donor identification, recruitment, and blood sample collection were done in collaboration with National Blood Service, Ghana. Clinical records (Supplementary File 1) of each prospective blood donor were taken to determine eligible blood donors. Eligibility variables were age, hemoglobin level, body weight, systolic pressure, and diastolic pressure. After a desirable volume of blood has been collected, 5 mL of anticoagulated blood sample was collected for *P. falciparum* screening.

### 2.5. Questionnaire Administration and Study Variables

Questionnaire was administered to each consented blood donor to elicit information on demographic parameters, *P. falciparum* transmission factors, and malaria control practices. Demographic information collected was age, gender, residential status, marital status, education level, occupation, and average monthly income. On *P. falciparum* transmission factors, donor cohabiting or sharing rooms with others, history of any of the room occupants suffering a bout of malaria in the past 3 months, living close to mosquito breeding sites, bedroom without ceiling, donor nocturnal outdoor activities for more than 2 hours, regular means of commuting, donor experiencing clinical malaria within past year, and frequent exposure to mosquito bites were assessed. Finally, availability of long-lasting insecticide treated nets (LLIN) in households, regular LLIN usage, indoor residual spraying with insecticides, usage of insecticide repellents, and frequent use of herbal preparations were malaria control practices assessed. Dependent variable was *P. falciparum* screening results (either positive or negative), while independent variables were the responses elicited by the questionnaires.

### 2.6. HIV, Syphilis, and Hepatitis B and C Donor Screening

Hepatitis B virus, hepatitis C virus, HIV I and II, and syphilis (*Treponema pallidum*) were screened using fourth-generation ELISA (Abnova, Taiwan). Screening was done according to manufacturer's instruction as previously described [14].

### 2.7. Detection of *P. falciparum* Specific Antigens (PfHRP2/pLDH) and Malaria Parasitemia

*P. falciparum* specific antigens (PfHRP2/pLDH) were detected in donor blood samples using SD Bioline PfHRP2/pLDH rapid diagnostic test kit (Gyeonggi-do, Republic of Korea). Screening was done according to the manufacturer's instructions. Screening for malaria parasites was done on the same day of sample collection. Infected donor blood units were quarantined and discarded subsequently. Malaria parasitemia was determined with 10% Giemsa staining. After staining for about 10 minutes, parasitemia was estimated by dividing the number of asexual parasites per at least 200 leukocytes and multiplied by estimated WBC of the patients/*μ*L of blood [16].

### 2.8. Selection of Comparative Groups

After the malaria screening, some of the donors with negative parasitological testing results were selected as comparative group. Because male donors were disproportionally more than females, a comparative group was selected to ensure there was no significant difference between the number of males and females that were infected and those that were not. To reduce bias to the barest minimum, the questionnaire data of noninfected individuals were compared to infected individuals in a ratio of 4 : 1, as recommended by Linden and Samuels [17].

### 2.9. Data Analysis

Chi-square test and logistic regression analyses were used to determine the level of association of independent variables with dependent variables. In all cases, *p* value of < 0.05 was considered statistically significant. Statistical analysis was done by SPSS version 24 (Chicago, IL, USA).

## 3. Ethical Approval

National Blood Service Ghana (NBSGRD/18903/01) and Ghana Health Service Ethical Review Committee (GHS-REC002/03/18) approved this study. Written informed consent was also sought from each prospective blood donor which included permission to publish the study outcome.

## 4. Results

### 4.1. Distributions of the Blood Donors among the Study Districts

**Figure 2:**
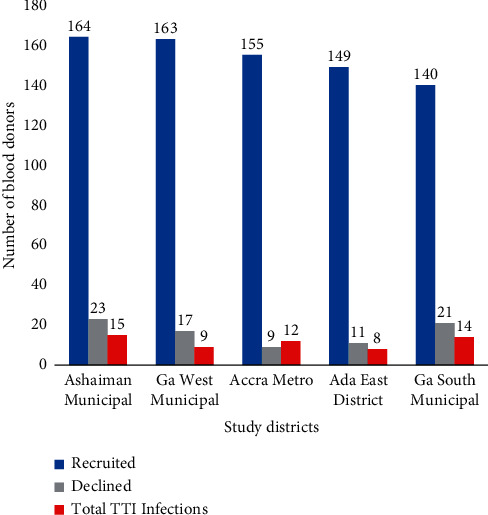
Number of blood donors successfully recruited and those that declined.

Using systematic sampling, a total of 910 prospective blood donors were eligible to partake in the study. Of these eligible blood donors, 81 (9.0%) declined participation for several reasons. Therefore, 829 eligible donors assented to take part in the study of which 58 (7.0%) were infected with various transfusion-transmitted infections (TTIs). The data of only apparently healthy blood donors (*n* = 771) were analyzed in this study. The distributions of the healthy blood donors were as follows: Ashaiman Municipal District 164 (21.3%), Ga West Municipal District 163 (21.1%), Accra Metro 155 (20.1%), Ada East 149 (19.2%), and Ga South 140 (18.2%) (Figure 2).

### 4.2. Prevalence of Transfusion-Transmitted Pathogens in the Blood Donors

A total of 58 (7.0%) of the 829 blood donors recruited into this study were infected with either HIV, syphilis, or hepatitis B or C virus. The prevalence of hepatitis C, HIV, syphilis, and hepatitis B was 8 (0.96%), 9 (1.1%), 18 (2.17%), and 23 (2.77%), respectively. There were no coinfections. Therefore, a total of 771 healthy blood donors, recruited from the five study districts, were analyzed in this study.

### 4.3. Comparative Prevalence of *P. falciparum* in Blood Donors in the Districts

The overall prevalence of *P. falciparum* in the study sites was 11.8%. The overall prevalence among males and females was 9.3% and 2.5%, respectively. The district with the highest prevalence of subclinical malaria among blood donors was Ga South (19.3%) (males and females were 14.3% and 5.0%, respectively) followed by Ada East (16.1% (males 12.7% and females 3.4%)), Ga West (12.3% (males 9.8% and females 2.5%)), Ashaiman (8.5% (males 6.7% and females 1.8%)), and Accra Metro (3.9% (males 3.9%)) (Figure 1).

### 4.4. Comparative Analysis of Demographic Characteristics for *P. falciparum* Infected and Noninfected Blood Donors

Table 1 represents the distribution of study variables among the study sites. Between the *P. falciparum* infected and the noninfected groups, gender categories (*p*=0.633) and marital status (*p*=0.116) were comparable. However, infected blood donors were significantly younger than noninfected donors (*p*=0.0041). Furthermore, infection status differed regarding residential status (*p* < 0.001), educational level (*p* < 0.001), occupation (*p* < 0.001), and monthly income (*p* < 0.001) (Table 2).

### 4.5. Association of Demographic Characteristics with Infection Status

Demographic features of the study participants associated with *P. falciparum* infection status are presented in Table 3. Logistic regression analysis indicated that infection status was not associated with gender nor marital status. However, compared with blood donors with tertiary education, the odds of being infected asymptomatically with *P. falciparum* were significantly higher if the donor has not had any formal education (OR = 6.12, 95% CI: 2.3–10.9, *p*=0.013) or has had only primary education (OR = 4.34, 95% CI: 2.0–9.4, *p*=0.035). Additionally, the odds of unemployed and student blood donors being infected asymptomatically with *P. falciparum* were 7.0 and 4.1 times higher compared to a donor being a government worker. Furthermore, blood donors with lower monthly income (less than 500 Ghana cedis or less than 100 USD) were at higher risk (OR = 3.7, 95% CI: 2.0–8.1, *p*=0.035) of having subclinical malaria compared to blood donors with high monthly incomes. Finally, rural dwelling blood donors were 9.1 times at higher risk of having subclinical malaria with reference to blood donors residing in urban communities.

### 4.6. Association of Transmission Factors with Infection Status

Malaria transmission factors associated with subclinical malaria are presented in Table 4. Binary regression analysis indicated that blood donors living close to mosquito breeding sites (OR = 8.8, 95% CI: 2.9–15.1), donors living in bedrooms with eaves (OR = 9.1, 95% CI: 3.0–18.1), those with prolonged (>2 hours) nocturnal activities (OR = 3.9, 95% CI: 1.2–10.1), and donors experiencing frequent exposure to mosquito bites (OR = 3.1, 95% CI: 1.1–5.3) have higher odds of subclinical malaria. Multivariate analysis further identified blood donors that use commercial vehicles frequently (OR = 2.5, 95% CI: 1.01–7.33) as having higher odds of subclinical malaria compared to donors that frequently use personal vehicles.

### 4.7. Association of Control Practices against Malaria with Infection Status

Malaria control practices significantly associated with subclinical malaria in blood donors identified in this study were not performing indoor residual spraying with insecticides (OR = 2.8, 95% CI: 1.74–5.21), not using insecticide repellants (OR = 4.4, 95% CI: 2.1–5.7), and frequent use of antimalarial herbal preparations (OR = 4.1, 95% CI: 2.6–6.71) (Table 5).

### 4.8. Algorithm for Determining Blood Donors with High Probability of Subclinical Malaria

**Figure 3:**
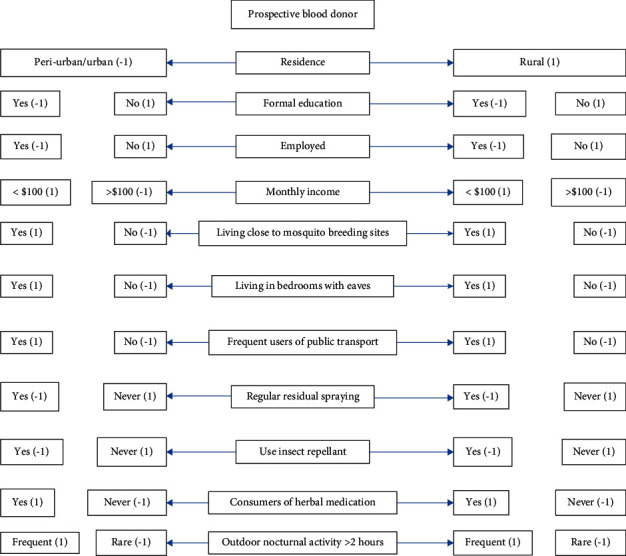
Algorithm for determining the accumulated risk of blood donors. Accumulated weight of <2: negligible risk; 3-4: tolerable risk; 5–8: undesirable risk; 9-10: intolerable risk.

Based on the independent variables found to be significantly associated with malaria parasitemia, an algorithm has been proposed to be used by blood banks to defer blood donors with high probability of subclinical malaria (Figure 3). Each significant variable was weighted one (1) point and its alternative response was weighted negative one (−1) point. Accumulation of the points determines the risk level of the donor. Using the Farmer criteria for risk assessment adopted by Bona et al. [18], accumulated risks <2, 3-4, 5–8, and >9 were classified as negligible risk, tolerable risk, undesirable risk, and intolerable risk, respectively.

### 4.9. Accumulative Weighted Points Based on the Developed Algorithm to Predict Malaria Parasitemia in Blood Donors

Table 6 represents the use of risk categorization and its corresponding accumulated weighted points to predict donor malaria parasitemia. Using the Farmer method for categorizing risks, up to 11 points could be obtained using the algorithm (Figure 3). Accumulated weighted point of less than 2 corresponded to 6.6% of donor malaria parasite antigenemia, while the same category cannot be used to predict malaria parasitemia. Donors with cumulative weighted points of 3 and 4 presented with tolerable risk. Among the blood donors, almost 10% of the donors within this category had PfHRP2 antigens detected in their blood, while 5.2% of the donors with malaria parasitemia were within the tolerable risk categorization. Furthermore, donor with accumulated weighted points of 5–8 presented with undesirable risk. Among the blood donors, 64.8% with PfHRP2 antigenemia had cumulative weighted point of 5–8, while 70.1% of the donors with parasitemia had accumulated weighted points of 5–8. Finally, according to the Farmer method of assessing risk, accumulated weighted points more than 8 are intolerable risk. Accumulative weighted points of greater than 8 were able to predict 18.7% of donors with PfHRP2 antigenemia, while 24.6% of malaria parasitemia could be predicted. Taken together, using accumulative weighted cut point of >5, the sensitivity of the algorithm to predict malaria parasitemia in blood donors was 94.7% (95% CI: 85.38–98.90%).

### 4.10. Specificity of the Algorithm

The proposed algorithm was tested on the blood donors without malaria. Of the noninfected comparative group (*n* = 364), 15 and 283 fell within the tolerable and the negligible risk groups, respectively. Among the rest (*n* = 66), 25 and 41 fell within the intolerable and the undesirable groups, respectively. Therefore, the algorithm was found to be 82% (95% CI: 77.5–85.7%) specific.

## 5. Discussion

This study reports exclusion criteria which can be used to defer blood donors with subclinical malaria. The following factors were found to be significantly associated with subclinical infection: residential status, educational level, occupation, and monthly income. Additionally, factors that aided the transmission of malaria were also analyzed. It was found that living close to mosquito breeding sites, living in bedrooms with eaves, and frequent users of public transport were significantly associated with subclinical malaria in the blood donors. Furthermore, the risk of parasite carriage was significantly higher among donors with habit of consuming antimalaria herbal preparations as well as donors that did not frequently performed indoor residual spraying with insecticides. Additionally, donors that did not use insecticide repellents and those that were nocturnally active for a long time as well as those that experienced frequent mosquito bites were associated with subclinical malaria. The factors identified in this study have previously been identified to be associated with clinical malaria [19–25]. These findings were not surprising since all clinical malaria cases begin asymptomatically. Therefore, factors associated with clinical malaria may also be associated with asymptomatic infections.

Blood donors that responded affirmatively to up to four of these associated factors have negligible or tolerable risk regarding *P. falciparum* carriage. Such donors can be bled for therapeutic use. However, a donor with accumulated weight of five or more should be tested for malaria before being made to donate blood. Those testing negative for malaria can be bled; this practice will eventually reduce the number of deferrals attributable to subclinical malaria. The algorithm was found to have relatively high false positive rate (18%). With this level of specificity, several donors without malaria could be deferred, as a result worsening the already existing low availability of blood for transfusion. Therefore, it is imperative that all donors with accumulated risk ≥5 be screened for malaria. With this algorithm, not all prospective donors will be screened but those within the high-risk zone. Another advantage of using this algorithm as a screening tool for malaria is cost reduction since only up to 18% of total blood donors would have to be tested for malaria.

Obviously, when blood donors suspected or with malaria are deferred, it will reduce availability of blood for clinical use. In this study, 7.0% (58/829) of donor blood units were rejected due to infection with either hepatitis C, HIV, syphilis, or hepatitis B in the donated blood. Considering the fact that 54 out of the 829 blood donors had malaria parasitemia, this would have further reduced blood availability by 6.5%. Due to this reason, deferring donors with malaria will aggravate the almost always unavailable blood situation in blood banks [26].

Notwithstanding the foregoing, malaria parasitemia in stored donor blood has been found to impact negatively on the quality of blood. Aninagyei et al. found severe storage lesions in stored blood with only detectable PfHRP2 antigens [14]. It could be inferred from that study that malaria rapid testing is enough to include or exclude a donor from bleeding. The study reported that storage of donor blood with detectable *P. falciparum* antigens affected all hematological cell lines. Significantly, red blood cells, hemoglobin, and haematocrit began to fall at week 1. Additionally, plasma hemoglobin elevation, increase in percentage of hemolysis above the permissible level of 0.8%, and potassium elevation were observed. In previous studies, excess in circulating hemoglobin was found to have negative impact on the intravascular nitric oxide metabolism [27]. Moreover, potassium is contraindicated in kidney failure patients [28]. In another study, elevation of circulating cytokines such as tumor necrosis factor alpha (TNF-*α*), interleukins (IL)-12 and IL-6 levels, and TNF-*α*/IL-10 ratio was observed in stored blood [29]. TNF-*α* and IL-6 are known to induce inflammatory through various mechanisms [30, 31]. Furthermore, high TNF-*α*/IL-10 ratio has also been associated with anaemia due to malaria in an earlier study [32]. Additionally, the negative effect of malaria parasitemia on platelets thrombogenicity has also been reported [33]. This plethora of metabolically active biomolecules has been suggested to induce either early or delayed transfusion reaction, which are mostly unexplained [34].

Obviously, the negative effect of malaria parasites on the quality of stored and fresh blood is overwhelming. It is in view of this that we propose an algorithm to be used to defer blood donors with subclinical malaria. The developed algorithm was able to detect almost 95% of the blood donors found to contain malaria parasites, while the sensitivity was 83.5% for donors detected by the rapid diagnostic test. With this algorithm, most donors with subclinical malaria could be detected. In as much as individuals infected with HIV and hepatitis B and C viruses may not donate blood almost for the rest of their lives, same could be applicable to an individual whose demographics place them in high-risk zone for malaria parasite infections.

## 6. Conclusions

This study proposes an algorithm to be used to exclude blood donors with subclinical malaria. The algorithm was developed based on demographics variables as well as malaria transmission and control practices that were found to be associated with blood donors with malaria. Out of the 57 blood donors found to contain malaria parasites by microscopy, this algorithm was able to detect almost 95% of them. We recommend the use of this algorithm to defer donors with malaria to prevent transfusion of malaria-infected blood to recipients with obvious clinical sequelae. Aside the sensitivity of the algorithm, it has an added advantage of predicting malaria in blood donors at no extra cost to blood centers.

## Figures and Tables

**Table 1:** Demographic characteristics of *P. falciparum* infected blood donors recruited from each study district.

	Ada East District		Ashaiman Municipal District		Accra Metro		Ga South Municipal District		Ga West Municipal District	
	Infected (*n* = 24)	Noninfected (*n* = 96)	Infected (*n* = 14)	Noninfected (*n* = 56)	Infected (*n* = 6)	Noninfected (*n* = 24)	Infected (*n* = 27)	Noninfected (*n* = 108)	Infected (*n* = 20)	Noninfected (*n* = 80)
Age years (mean ± SD)	25.7 ± 6.3	33.7 ± 6.9	23.0 ± 4.1	35.0 ± 5.9	25.8 ± 5.7	29.8 ± 8.3	25.9 ± 3.9	32.1 ± 6.8	27.2 ± 5.0	31.1 ± 6.9
Gender										
Male *n* (%)	19 (79.2)	77 (80.2)	11 (78.6)	49 (87.5)	6 (100)	21 (87.5)	20 (74.1)	85 (78.7)	16 (80.0)	64 (80.0)
Female *n* (%)	5 (20.8)	19 (19.8)	3 (21.4)	7 (12.5)	0 (0.0)	3 (12.5)	7 (25.9)	23 (21.3)	4 (20.0)	16 (20.0)
Residential status										
Rural *n* (%)	13 (54.2)	8 (8.3)	5 (35.7)	0 (0.0)	3 (50.0)	0 (0.0)	14 (51.9)	11 (10.2)	13 (65.0)	6 (7.5)
Periurban *n* (%)	7 (29.2)	22 (22.9)	6 (42.9)	18 (32.1)	2 (33.3)	14 (58.3)	9 (33.3)	41 (37.9)	4 (20.0)	33 (41.3)
Urban *n* (%)	4 (16.7)	66 (68.8)	3 (21.4)	38 (67.9)	1 (16.7)	10 (41.7)	4 (14.8)	56 (51.9)	3 (15.0)	41 (51.3)
Marital status										
Single *n* (%)	7 (29.2)	31 (32.3)	8 (57.1)	24 (42.8)	4 (66.7)	10 (41.7)	5 (18.5)	56 (51.9)	6 (30.0)	34 (42.5)
Married *n* (%)	11 (45.8)	52 (54.2)	4 (28.6)	26 (46.4)	2 (33.3)	7 (29.2)	13 (48.1)	38 (35.2)	11 (55.0)	37 (46.3)
Divorced *n* (%)	4 (16.7)	7 (7.3)	1 (7.1)	4 (7.1)	0 (0.0)	4 (16.7)	6 (22.2)	8 (7.4)	2 (10.0)	4 (5.0)
Widow/widower *n* (%)	2 (8.3)	6 (6.3)	1 (7.1)	2 (3.6)	0 (0.0)	3 (12.5)	3 (11.1)	6 (5.6)	1 (5.0)	5 (6.3)
Education level										
No formal education *n* (%)	7 (29.2)	8 (8.3)	2 (14.2)	0 (0.0)	2 (33.3)	1 (4.2)	3 (11.1)	10 (9.3)	2 (10.0)	3 (3.8)
Primary education *n* (%)	2 (8.3)	9 (9.4)	3 (21.4)	0 (0.0)	0 (0.0)	1 (4.2)	5 (18.5)	9 (8.3)	7 (35.0)	7 (8.8)
Junior high school *n* (%)	6 (25.0)	17 (17.7)	3 (21.4)	13 (23.2)	2 (33.3)	2 (8.3)	4 (14.8)	16 (14.8)	4 (20.0)	19 (23.8)
Senior high school *n* (%)	3 (12.5)	24 (25.0)	1 (7.1)	18 (32.1)	1 (16.7)	3 (12.5)	5 (18.5)	20 (18.5)	3 (15.0)	31 (38.8)
Tertiary *n* (%)	6 (25.0)	38 (39.6)	5 (35.7)	25 (44.6)	1 (16.7)	17 (70.8)	10 (37.0)	53 (47.1)	4 (20.0)	20 (25.0)
Occupation										
Unemployed *n* (%)	3 (12.5)	7 (7.3)	3 (21.4)	0 (0.0)	2 (33.3)	2 (8.3)	3 (11.1)	5 (4.6)	3 (15.0)	2 (2.5)
Students *n* (%)	3 (12.5)	6 (6.3)	0 (0.0)	0 (0.0)	1 (16.7)	1 (4.2)	4 (14.8)	3 (2.8)	2 (10.0)	2 (2.5)
Government workers *n* (%)	5 (20.8)	29 (30.2)	2 (14.2)	11 (19.6)	0 (0.0)	5 (20.8)	6 (22.2)	41 (37.9)	3 (15.0)	13 (16.3)
Petty trading *n* (%)	8 (33.3)	25 (26.0)	6 (42.9)	18 (32.1)	2 (33.3)	9 (37.5)	9 (33.3)	40 (37.0)	7 (35.0)	30 (37.5)
Private sector workers *n* (%)	5 (20.8)	29 (30.2)	3 (21.4)	27 (48.2)	1 (16.7)	7 (29.2)	5 (18.5)	19 (17.6)	5 (25.0)	33 (41.3)
Average monthly income										
0–500 (0–100) *n* (%)	6 (25.0)	13 (13.5)	5 (35.7)	9 (16.1)	1 (16.7)	0 (0.0)	6 (22.2)	24 (22.2)	7 (35.0)	20 (25.0)
501–1000 (100.2–200) *n* (%)	4 (16.7)	19 (19.8)	2 (14.2)	17 (30.3)	2 (33.3)	9 (37.5)	5 (18.5)	40 (37.0)	4 (20.0)	18 (22.5)
1001–2000 (200.2–400) *n* (%)	6 (25.0)	12 (12.5)	3 (21.4)	15 (26.8)	0 (0.0)	5 (20.8)	8 (29.6)	16 (14.8)	4 (20.0)	22 (27.5)
>2000 (>400) *n* (%)	2 (8.3)	39 (40.6)	1 (7.1)	15 (26.8)	0 (0.0)	7 (29.2)	1 (3.7)	20 (18.5)	0 (0.0)	16 (20.0)

Average monthly income: Ghana cedi (Gh¢) (US dollar) ($^∗^) based on 2018 exchange rate of 1 US$: 5 Ghana cedis.

**Table 2:** Association of study variables with *P. falciparum* infection status.

Variable	Infected (*n* = 91)	Noninfected (*n* = 364)	Test statistic	*p* value
Age (years) (mean ± SD)	25.32 ± 4.3	32.19 ± 7.7	2.03	0.041^a^
Gender			0.22	0.633^b^
Male *n* (%)	72 (79.1)	296 (81.3)		
Female *n* (%)	19 (20.9)	68 (18.7)		
Residential status			121.14	*p* < 0.001^b^
Rural *n* (%)	48 (52.7)	25 (6.9)		
Periurban *n* (%)	28 (30.7)	128 (35.2)		
Urban *n* (%)	15 (16.5)	211 (57.9)		
Marital status			5.89	0.116^b^
Single *n* (%)	30 (32.9)	155 (42.6)		
Married *n* (%)	41 (45.1)	160 (43.9)		
Divorced *n* (%)	13 (14.3)	27 (7.4)		
Widow/widower *n* (%)	7 (7.7)	22 (6.0)		
Education level			29.89	*p* < 0.001^b^
No formal education *n* (%)	16 (17.6)	22 (6.0)		
Primary education *n* (%)	17 (18.7)	26 (7.1)		
Junior high school *n* (%)	19 (20.9)	67 (18.4)		
Senior high school *n* (%)	13 (14.3)	96 (26.4)		
Tertiary *n* (%)	26 (28.6)	153 (42.0)		
Occupation			26.29	*p* < 0.001^b^
Unemployed *n* (%)	14 (15.4)	16 (4.4)		
Students *n* (%)	10 (10.9)	12 (3.3)		
Government workers *n* (%)	16 (17.6)	99 (27.2)		
Petty trading *n* (%)	32 (35.2)	122 (33.5)		
Private sector workers *n* (%)	19 (20.9)	115 (31.6)		
Average monthly income				
0–500 (0–100) *n* (%)	25 (37.3)	66 (19.6)	22.67	*p* < 0.001^b^
501–1000 (100.2–200) *n* (%)	17 (25.4)	103 (30.7)		
1001–2000 (200.2–400) *n* (%)	21 (31.3)	70 (20.8)		
>2000 (>400) *n* (%)	4 (5.9)	97 (28.9)		

^a^T-test; ^b^chi-square.

**Table 3:** Bivariable logistic regression analysis identifying factors associated with *P. falciparum* infection in blood donors.

Factors	Categories	*N*	*n* (%) infected	*p* value	OR	OR (95% CI)
Educational level	No formal education	38	16 (44.7)	0.013	6.12	2.3–10.9
	Primary education	43	17 (44.2)	0.035	4.34	2.0–9.4
	Junior high school	86	19 (18.6)	0.115	1.49	0.68–3.41
	Senior high school	109	13 (11.9)	0.083	0.63	0.4–1.1
	Tertiary	179	26 (13.5)		1	

Occupation	Unemployed	30	14 (46.7)	<0.001	7.0	3.9–17.2
	Students	22	10 (45.5)	0.04	4.1	1.9–14.7
	Petty trading	154	32 (20.8)	0.13	0.73	0.15–3.62
	Private sector workers	134	19 (14.2)	0.70	0.16	0.01–1.73
	Government workers	115	16 (13.9)		1	

Monthly income	0–500 (0–100)	91	25 (27.5)	0.035	3.7	2.0–8.1
	501–1000 (100.2–200)	120	17 (14.1)	0.068	0.70	0.1–3.8
	1001–2000 (200.2–400)	91	21 (23.1)	0.169	0.39	0.11–1.47
	>2000 (>400)	101	4 (3.8)		1	

Residential status	Rural	73	48 (65.7)	<0.001	9.1	3.5–27.3
	Periurban	156	28 (17.9)	0.13	2.2	0.97–10.2
	Urban	226	15 (6.6)		1	

Monthly income: Gh¢ ($^∗^) (US dollar) based on 2018 exchange rate of 1 US$: 5 Ghana cedis.

**Table 4:** Logistic regression analysis identifying transmission factors associated with *P. falciparum* infection in blood donors.

Transmission factor	Response	*N*	*n* (%) infected	*p* value	OR	OR (95% CI)
Cohabitation/shared room	Yes	354	68 (19.2)	0.215	0.73	0.11–3.0
	No	101	23 (22.8)		1	

History of roommate(s) with malaria in past 3 months	Yes	14	3 (21.4)	0.115	1.01	0.23–6.1
	No	441	88 (20.0)		1	

Living close to mosquito breeding sites	Yes	136	55 (40.4)	<0.001	8.8	2.9–15.1
	No	319	36 (11.3)		1	

Bedroom without ceiling	Yes	15	9 (60.0)	<0.001	9.1	3.0–18.1
	No	440	82 (18.6)		1	

Staying outdoor at night > than 2 hours	Yes	97	43 (44.3)	0.008	3.9	1.2–10.1
	No	358	48 (13.4)		1	

Regular means of commuting	Self-driven vehicle	131	29 (22.1)		1	
	Public vehicle	98	31 (31.6)	0.034	2.5	1.01–7.33
	Walking	98	10 (10.2)	0.222	0.67	0.03–1.31
	Riding	128	21 (16.4)	0.105	0.88	0.11–2.02

Clinical malaria within past year	Yes	81	22 (27.1)	0.09	1.3	0.45–4.2
	No	374	69 (18.4)		1	

Frequent exposure to mosquito bites	Yes	47	16 (34.0)	0.011	3.1	1.1–5.3
	No	408	75 (18.4)		1	

**Table 5:** Bivariable logistic regression analysis for transmission factors associated with subclinical malaria in blood donors.

Transmission factor	*N*	*n* (%) infected	*p* value	OR	OR (95% CI)
Availability of LLIN in household					
Yes	260	39 (15.0%)	0.093	0.7	0.46–1.13
No	195	52 (26.7%)		1	
Regular LLIN usage					
Yes	61	11 (18.0%)	0.215	1.6	0.2–3.1
No	199	28 (14.1%)		1	
Indoor residual spraying with insecticides					
Yes	28	5 (17.6%)		1	
No	427	86 (20.1%)	0.013	2.8	1.74–5.21
Usage of insecticide repellents					
Yes	214	17 (8.0%)		1	
No	241	74 (30.7%)	<0.001	4.4	2.1–5.7
Frequent use of antimalarial herbal preparations					
Yes	261	59 (22.6%)	0.033	4.1	2.6–6.71
No	194	32 (16.5%)		1	

OR: odds ratio; LLIN: long-lasting insecticide treated nets.

**Table 6:** *P. falciparum* exposure risk categorization of the blood donors.

		RDT Positive (*n* = 91)		Microscopy (*n* = 57)		Noninfected	
Risk categorization	Weighted point (range)	Frequency	Percent (%)	Frequency	Percent (%)	Frequency	Percent (%)
Negligible risk	≤2	6	6.6	0	0.0	279	76.6
Tolerable risk	3-4	9	9.9	3	5.2	59	16.2
Undesirable risk	5–8	59	64.8	40	70.1	15	4.1
Intolerable risk	>8	17	18.7	14	24.6	11	3.0
Deferral	≥5	76	83.5	54	94.7	26	7.1

## Data Availability

Data obtained in this study have been presented in this publication.

## Abbreviations

LLIN: Long-lasting insecticide treated net
OR: Odds ratio
PfHRP2: *Plasmodium falciparum* histidine rich protein 2
pLDH: *Plasmodium* lactate dehydrogenase
TTM: Transfusion-transmitted malaria.

## Supplementary Materials

Supplementary Materials Donor clinical questionnaire used to determine the eligibility of Ghanaian blood donors (source: National Blood Service, Ghana).
