# Supplementary material for: The Use of Screening Algorithm to Defer Blood Donors with Subclinical Malaria
Source: J Trop Med. 2021 Aug 13;2021:9942721. doi: 10.1155/2021/9942721 (PMC8380175; doi:10.1155/2021/9942721)
Supplement: Supplementary Materials — Donor clinical questionnaire used to determine the eligibility of Ghanaian blood donors (source: National Blood Service, Ghana). [file 9942721.f1.pdf]

4. Health Questionnaire

Blood donation should be safe for both the donor and the eventual recipient of the blood donated. The following questions will help us determine whether it is safe for YOU to donate blood today, and whether the blood is likely to be safe enough to give to a sick person. We cannot rely entirely on laboratory tests, as they may not always be able to detect infectious agents and other problems, so please answer the following questions TRUTHFULLY. Thank you!

Please check "✓" in the boxes and qualify any 'Yes' response by underlining the specific item that applies.

1. Are you not feeling well today, i.e. fever, cough, headache or cold? Yes ☐ No ☐
2. Have you ever been deferred as a blood donor or told not to donate blood? Yes ☐ No ☐
   If Yes, for what reason?____
3. Are you taking drugs for high blood pressure, diabetes, or asthma? Yes ☐ No ☐
4. Do you have a history of epilepsy, stomach ulcer, or tuberculosis? Yes ☐ No ☐
5. Have you taken medication or been vaccinated in the last 4 weeks? Yes ☐ No ☐
   If Yes, please give details ____
6. Have you had jaundice, liver disease, or a positive blood test for hepatitis? Yes ☐ No ☐
7. Do you have sickle cell disease, e.g. SS, SC, and Sßthal? Yes ☐ No ☐
8. Have you ever taken self-injected drugs or been diagnosed as a drug addict? Yes ☐ No ☐
9. Have you in the last 12 months had an injection in a place that is not a hospital or clinic; skin scarring/tattoo or cutting by traditional healer, including circumcision? Yes ☐ No ☐
10. Have you had dental treatment in the last 1 week? Yes ☐ No ☐
11. Have you in the past 6 months had a major surgical operation? Yes ☐ No ☐
12. Have you in the last 6 months received blood or blood product transfusion? Yes ☐ No ☐
13. Have you in the last 6 months lost more than 5kgs due to illness? Yes ☐ No ☐
14. Have you in the last 6 months had unprotected sex with more than one partner or have paid/been paid to have sex? Yes ☐ No ☐
15. Have you ever had gonorrhoea or other sexually transmitted disease? Yes ☐ No ☐
16. Have you in the last 6 months had sex with a homosexual? Yes ☐ No ☐
17. Have you or your partner ever tested positive for HIV (AIDS) or hepatitis? Yes ☐ No ☐
18. After blood donation, are you going to take part in any vigorous activity, such as climbing, driving a heavy vehicle or work at hazardous or heights? Yes ☐ No ☐
19. Are you pregnant, breast-feeding or had a miscarriage in the past 12 months? Yes ☐ No ☐

If the donor answers 'YES' to any of these questions refer to the *"Donor Selection Criteria Guide for Donor Attendants*" in the Donor Selection Manual to determine if the donor can be accepted.

Donor Declaration

I confirm that the information I have provided regarding my current state of health, previous illnesses, medication history, and sexual health are TRUE and CORRECT to the best of my knowledge. I understand my blood will be tested for HIV, Hepatitis B, Hepatitis C and Syphilis and I have no reason to believe I am a carrier of any. I understand that any incorrect answer to the question above may harm my health or that of a person who will receive my blood. I promise to notify the Blood Service/Blood Bank of any change to the information I have provided as soon as I am aware of it.

....

Donor's Signature Counsellor's Signature Date

Form NBTS/DN 20 v2

National Blood Transfusion Service
Donor Clinical Record

Session Number ________________ Date __________________ Venue ______________________________

1. Personal Data

1.1 First Name __________________________________ 1.2 Last Name ____________________________

1.3 Date of Birth ________________________________ 1.4 Sex ☐ Male ☐ Female

1.5 Address (Residence) ____

1.6 Address (Workplace) ____

1.7 Occupation ____

1.8 Phone Number ______________________________ 1.9 Email ________________________________

2. Voluntary Donors Only

2.1 Have you donated blood before? ☐ Yes ☐ No 2.2 If Yes, when was the last time? ________________

2.3 How many times as Voluntary ________ Replacement __________ 2.4 Donor Card # ___________

3. Replacement/Family Donors Only

3.1 Name of Patient ____

3.2 Hospital ___________________________________ 3.3 Ward ___________________________________

3.4 Your relationship to Patient ____________________ 3.5 How many times have you donated? _______

Clerking Officer.. Signature..

Section 4 ➔

Office Use Only

5. Donor Selection

5.1 Appearance: ☐ Passed ☐ Failed 5.2 Medical History: ☐ Passed ☐ Failed

5.3 Wt _____________ kg 5.4. Blood Pressure______________mmHg 5.5 Pulse _____________bpm

5.6 Hb by CuSO4 ☐ Passed ☐ Failed 5.7 Hb checked _________________________g/dL

5.8. HBsAg ____________________________________ 5.9. HCV ___________________________________

5.10 Outcome of Screening:

☐ Qualifies to Donate ☐ Disqualified ☐ Deferred for _____________ (duration)

Comments: ____

Name of Nurse.. Signature..

6.Blood Collection

6.1 Donation Number: [ ]

6.2 Outcome of Phlebotomy: ☐ Successful

☐ Unsuccessful ☐ Underbled ☐ Venous Access ☐ Donor Reaction

Name of Nurse.. Signature..
